# Antioxidant/Anti-Inflammatory Potential and Sensory Acceptance of Granola Bars Developed with Sorghum Sprout Flour Irradiated with UV-A LED Light

**DOI:** 10.3390/foods14101787

**Published:** 2025-05-17

**Authors:** Alan A. Ruiz-Hernández, Ofelia Rouzaud-Sández, Maribel Valenzuela-González, J. Abraham Domínguez-Avila, Gustavo A. González-Aguilar, Maribel Robles-Sánchez

**Affiliations:** 1 Departamento de Investigación y Posgrado en Alimentos, Universidad de Sonora, Blvd. Luis Encinas y Rosales, Colonia Centro, Hermosillo 83000, Sonora, Mexico; alanamado12@hotmail.com (A.A.R.-H.); ofelia.rouzaud@unison.mx (O.R.-S.); maribelvalezuelag4@gmail.com (M.V.-G.); 2 SECIHTI-Centro de Investigación en Alimentación y Desarrollo, A.C., Carretera Gustavo Enrique Astiazarán Rosas, No. 46, Colonia La Victoria, Hermosillo 83304, Sonora, Mexico; abrahamdominguez9@gmail.com; 3 Centro de Investigación en Alimentación y Desarrollo, A.C., Carretera Gustavo Enrique Astiazarán Rosas, No. 46, Colonia La Victoria, Hermosillo 83304, Sonora, Mexico; gustavo@ciad.mx

**Keywords:** functional foods, emerging technologies, obesity, sensory evaluation, nitric oxide, anti-inflammatory

## Abstract

Overweight and obesity are worldwide problems; several strategies have been applied to counteract them, including the development of functional foods with specific bioactivities. Sorghum has been shown in in vitro and in vivo studies to improve various biomarkers related to overweight, obesity, and inflammation, particularly when sprouted and irradiated with UV light. In the present study, irradiated sorghum sprouts were used to prepare granola bars; their phenolic profile, antioxidant activity, in vitro bioaccessibility, anti-inflammatory potential, and sensory acceptability were measured. Gallic acid increased in response to irradiation, while catechin increased in response to sprouting. In vitro digestion showed higher intestinal recovery of phenolics and antioxidant capacity, as well as a significant decrease in nitric oxide content, an inflammation biomarker. A sensory analysis showed scores of approximately 5.5–6.5 (on a 9-point hedonic scale) for most variables analyzed, suggesting adequate acceptability. Sorghum bars made with irradiated sorghum sprouts present high potential as health foods that could help counteract the problems of overweight, obesity, and related diseases. Further in vitro and in vivo studies are needed to demonstrate the benefits of consuming this food.

## 1. Introduction

Although the idea of treating obesity as an independent disease is still controversial, it is certain that this condition is related to several metabolic diseases [1]. Several strategies and joint efforts to address the problem of obesity have been applied worldwide, including innovative technology for the development of new foods [2].

Among the new trends, functional food production is being driven by technological advances, changes in consumer preferences, trends in plant-based foods [3], functional foods for mental health [4], functional and innovative ingredients such as microalgae and fungi [5,6], and functional foods for immunity [7]. Among these trends, sorghum (*Sorghum bicolor* (L.) *Moench*) is the fifth most produced cereal worldwide, used for fodder and biofuel production [8], and is a versatile and nutritious cereal that has gained attention in recent years due to its multiple beneficial qualities for health, agriculture, and the environment [9]. Sorghum has nutrient-rich [10], gluten-free [11], antidiabetic [12], antihypertensive [13], anti-inflammatory [14], and anticancer [15,16] properties, as well as qualities for the prevention and treatment of Alzheimer’s disease [17], and these characteristics make it stand out from other cereals. To improve these qualities, different technologies are applied to sorghum, including extrusion [18], microwaves [19], and pulsed electric fields [20]. Within this group, sprouting is widely used in grains, in which different physiological and biochemical changes occur that have effects on macro- and micronutrients and bioactive compounds [21], which can be obtained at low costs, is simple, and is environmentally friendly.

In addition to the increase in antioxidant potential that occurs during sprouting, there are treatments that can further increase it. Among them, ultrasound [22] and microwaves [23] have been used. Likewise, different light spectra have been used in some seeds to assess their effect on the synthesis of different bioactive compounds [24,25].

Among light, ultraviolet light is divided into UV-A (320–400 nm), UV-B (280–320 nm), and UV-C (100–280 nm), and unlike other technologies, ultraviolet light emitted by light-emitting diodes (LEDs) has greater advantages such as robustness, energy efficiency, long life, wave specificity, and good temporal stability [26,27]. It has been documented that UV-A light in plants is mediated by photoreceptors such as UVR-8, which is involved in gene expression and photomorphogenic responses, including the induction of phenolic compound synthesis, among others [28]. This mechanism has been utilized to enhance the antioxidant potential of plants and potential foods with beneficial health effects [29].

Sorghum has demonstrated great potential for the development of novel foods; for example, cookies with a low glycemic index have been developed in the Ivory Coast to treat diabetes [30], or mangosteen-fortified sorghum cookies with potential antidiabetic effects have been produced in Indonesia [31]. Therefore, based on trends in food development and strategies for counteracting overweight, obesity, and metabolic diseases, the development of a granola bar enriched with sorghum sprouts irradiated with UV-A LED with potential antioxidant and anti-inflammatory effects is proposed.

## 2. Materials and Methods

### 2.1. Sorghum Sprouting

White sorghum seeds were subjected to sanitation using a 0.1% sodium hypochlorite solution at a 1:3 (*w*/*v*) ratio for 5 min. Subsequently, the disinfectant was removed, and the seeds were thoroughly rinsed with distilled water. The sanitized seeds were germinated for 28 h at 35 °C and 98% relative humidity with a light–dark photoperiod of 8/16 h in a germinator of our own construction. At the end of germination sensu stricto, the sample was left to sprout for 36 h. Subsequently, irradiation was applied to the growing shoots at 5.1 µW/cm^2^ (UV-A LED 395 nm) (RR-ELECTRONICS^®^, Tamil Nadu, India) for 11.9 h [32]. The resulting sprouts were frozen at −20 °C (Criotec^®^, Chivasso, Italy), dried at 45 °C (Felisa^®^, Zapopan, Jalisco, Mexico), and milled (Semillas de vida^®^, Cuernavaca, Morelos, Mexico) until an irradiated sorghum sprout flour (ISSF) was obtained.

### 2.2. Granola Bar Preparation

The formulation from Zamora-Gasga et al. [33], with modifications, was used to prepare a granola bar. Oat flakes, coconut oil, agave honey, cashews, almonds, and coconut were used to prepare each granola bar (Table 1). Different flours were used to prepare different bars, using sorghum flour (SF), oat flour (OF), sorghum sprout flour (SSF), and ISSF. The proportions of the different flours used were chosen based on internal preliminary studies, where different sensory characteristics were evaluated. Each recipe was hand mixed, molded, and baked at 165 °C for 15 min. The phenolic profile, antioxidant activity, in vitro bioaccessibility, anti-inflammatory potential, and sensory acceptability of each bar were measured.

### 2.3. Obtaining Free and Bound Compounds

For the extraction and quantification of phenolic compounds, the bars were ground at 28,000 rpm for 5 min in a grain mill (Semillas de vida^®^) and homogenized.

To obtain the free phenolic fraction of the samples, extraction with methanol was used. In conical tubes (15 mL), 1 g of (defatted) sample was weighed, 15 mL of 80% methanol (JT Baker, Mexico City, Mexico) was added, and the mixture was sonicated for 1 h at 50–60 Hz in cold conditions (Branson 2510^®^, Danbury, CT, USA). The sample was centrifuged at 4000 rpm, the supernatant was recovered using a Whatman grade 1 filter and centrifuged at 4000 rpm, and the final supernatant was recovered using a Whatman grade 1 filter. The final supernatant was evaporated under vacuum (Rotavapor^®^ R100, Buchi, Flawil, Switzerland) at 45 °C until dryness and resuspended in 50% methanol. The process was repeated 2 times, and the resulting supernatants were evaporated under vacuum and resuspended in 50% methanol [34].

To obtain the conjugated compounds of the food matrix, 1 mL of the resuspended methanol extraction was mixed with 5 mL of NaOH (2 N degassed, Sigma-Aldrich, St. Louis, MO, USA). The air was displaced with nitrogen, and the mixture was sonicated for 1 h, after which the pH was adjusted to 1.5–2.0 with 6 N HCl. Then, ethyl acetate (7 mL) was added, and the mixture was stirred for 2 min. For phase separation, the mixture was centrifuged at 4000 rpm for 15 min, and this step was repeated 2 times. Ethyl acetate (Sigma-Aldrich) was recovered and evaporated under vacuum in a rotary evaporator, and the sample was resuspended in 7 mL of 50% methanol [35]. For the bound fraction, 100 mg of dry sample (45 °C) was obtained from the solid residue of the methanol fraction and mixed with 5 mL of NaOH (2 N, degassed). The air was displaced with nitrogen, and the mixture was sonicated for 3 h. The extraction continued according to the methodology used for conjugated compounds. The samples were defatted before extraction according to the method of López-Bascón et al. [36], with slight modifications.

### 2.4. Determination of the Total Phenol Content

Total phenolic compounds were quantified via the Folin–Ciocalteu assay [37] using a FLUOStar Omega microplate reader v 1.20 (BMG Labtech Inc., Ortenberg, Germany) with modifications. For each extract, 30 µL was mixed with 150 µL of Folin–Ciocalteu reagent (Sigma-Aldrich) diluted 1:9 with deionized water. Then, 120 µL of sodium carbonate (Sigma-Aldrich) solution (0.075 mg/mL) was added, and the mixture was incubated for 60 min in the dark. Changes in absorbance were monitored at 765 nm against a reagent blank. A concentration curve with gallic acid (Sigma-Aldrich) as a standard was used. The results are expressed as milligram gallic acid equivalents (mg GAE) per gram of sample (mg GAE/g).

### 2.5. Antioxidant Capacity

#### 2.5.1. 1,1-Diphenyl-2-Picrylhydrazyl (DPPH)

The DPPH assay evaluates the ability to scavenge the DPPH radical (Sigma-Aldrich), detected via a change in absorbance at 515 nm. Briefly, mixtures were prepared in microplate wells by combining 280 µL of a methanolic solution of DPPH (0.025 mg/mL) with 20 µL of each extract. The samples were subsequently incubated in the dark at room temperature for 60 min. The absorbance was measured at 515 nm, and changes in the DPPH radical scavenging capacity were recorded. A standard Trolox (Sigma-Aldrich) curve was used, and the results were expressed in micromolar Trolox equivalents per gram of sample (µM TE/g) [38].

#### 2.5.2. Trolox Equivalent Antioxidant Capacity (TEAC)

The color change at 715 nm that is produced by the ability of antioxidant molecules to scavenge the ABTS^●+^ cation radical (Sigma-Aldrich) was evaluated [39]. Potassium persulfate (Sigma-Aldrich, 17.6 µL of a 37.8 mg/mL solution) was used for the activation of the ABTS radical (3.86 mg/mL), which was incubated for 16 to 18 h at room temperature. A working solution was prepared by mixing 1 mL of the ABTS solution with ≈88 mL of ethanol and adjusted to an absorbance of 0.70 ± 0.02. For the assay, in microplate wells, 280 µL of the working solution was mixed with 20 µL of each of the extracts and incubated for 5 min. The results are expressed as µM TE/g, according to a standard curve.

### 2.6. Quantification of Phenols via Ultra-High-Performance Liquid Chromatography Diode Array Detection (UHPLC-DAD)

The components were separated using an Agilent 1290 Infinity Binary LC system to evaluate the phenolic profile. A Zorbax Eclipse Plus C18 column (2.1 × 50 mm, 1.8 µm, Agilent, Santa Clara, CA, USA) at 30 °C was used. For the mobile phase, a linear gradient of 0.1% formic acid in water (solvent A) and 0.1% formic acid in acetonitrile (solvent B) (JT Baker) was used as follows: 97–93% A, 0–3 min; 93–90% A, 3–5 min; 90–88% A, 5–8 min; 88–85% A, 8–10 min; 85–85% A, 10–15 min; 85–45% A, 15–18 min; and 45–10% A, 18–20 min. The injection volume was 10 μL at a flow rate of 0.4 mL/min. The retention times and spectra of each compound were evaluated for identification [40].

### 2.7. In Vitro Digestion

To simulate gastrointestinal digestion conditions, the protocol of Salazar-López et al. [41] was used, through which the bioaccessibility of free phenols, antioxidant recovery, and individual phenolic compounds were evaluated for samples from the different granola bars. Briefly, for the oral phase, asymptomatic fasting volunteers (1 per sample) chewed 1 g of the sample. After this phase, the volunteers expelled the liquid and rinsed their mouths twice with 5 mL of distilled water. The samples used in the oral phase were frozen at −20 °C, and the remaining samples were stored for the subsequent phases. In the gastric phase, 0.2 M HCl-KCl buffer was subsequently added to the sample, which was subjected to the oral phase at pH 1.5. Next, 700 µL of pepsin solution (300 mg/mL) from porcine gastric mucosa (Cat. P7000 powder ≥ 250 units/mg solid, Sigma-Aldrich) was added to reach a final enzyme concentration of 2000 U/mL. The gastric digesta was incubated in a water bath at 37 °C and 100 rpm for 1 h. At the end of the incubation period, the samples were frozen, and the remaining samples were saved for the intestinal phase. In this phase, phosphate buffer (0.1 M) was first added at pH 7.5. Next, 1 mL of pancreatin (17 mg/mL) from porcine pancreas (Cat. P1750 4× USP specifications, Sigma-Aldrich) was added to reach a final enzyme concentration of 100 U/mL (trypsin activity), and bile salts (80 mg) (Cat. B8756, Sigma-Aldrich) were added. The samples were shaken at 37 °C and 100 rpm for 6 h. After incubation, the oral, gastric, and intestinal samples were frozen at −20 °C, lyophilized (LABCONCO^®^ freeze dryer at −50 °C for 3 days), and resuspended in 50% methanol for subsequent analysis. Blanks followed the same protocol but without samples. Bioaccessibility was expressed as a percentage of the total free phenols:

Bioaccessibility (%) = (Total free phenol content of the intestinal fraction/total phenols (free + bound + conjugated) of nondigested samples) × 100(1)

DPPH and TEAC values were also analyzed via this procedure and expressed as the percentage of antioxidant activity recovery (%).

### 2.8. Anti-Inflammatory Capacity

#### 2.8.1. Cell Culture

Mouse RAW 264.7 macrophages from the American Culture Collection (ATCC) were used and cultured in Dulbecco’s Modified Eagle Medium (DMEM) supplemented with 10% fetal bovine serum, sodium bicarbonate (3.5 g/L), an antimycotic antibiotic (1%), and sodium pyruvate (110 mg/L) (Sigma-Aldrich). The culture mixture was placed in a 75 cm^2^ flask at 37 °C and 5% CO_2_ in a humidified atmosphere, as described by Salazar López et al. [34].

#### 2.8.2. Effect of Extracts on Cell Viability

RAW 264.7 cells were cultured at 37 °C and 5% CO_2_ for 24 h in 96-well COSTAR plates (1 × 10^4^ cells/well). After incubation, the medium was replaced with fresh medium containing different concentrations of food extracts, and the intestinal fraction digests (25, 12.5, and 6.25 µg/mL) were resuspended in <1% dimethyl sulfoxide (DMSO, Sigma-Aldrich). The culture was incubated for 24 h, after which the medium containing the treatments was removed. Subsequently, 20 µL of 3-(4,5-dimethylthiazol-2-yl)-2,5-diphenyltetrazolium bromide (MTT, Sigma-Aldrich) dissolved in the medium was added to the adherent cells and incubated again for 2 h. As a result, MTT was transformed to formazan, which was spectrophotometrically assessed at 570 nm [34]. Cell viability (CV) was calculated according to the following equation:

CV (%) = (sample absorbance/absorbance control cells) × 100.(2)

#### 2.8.3. Determination of Nitric Oxide (NO) Production

To measure nitric oxide (NO) production, RAW 264.7 cells (2.5 × 10^5^) were cultured in 96-well plates at 37 °C and 5% CO_2_ for 24 h. Subsequently, medium containing lipopolysaccharide (LPS, 1 μg/mL, Sigma-Aldrich) was added to induce nitric oxide production, and treatments (extracts and food digests) were added at concentrations of 25, 12.5, and 6.25 µg/mL. NO production in the culture medium was indirectly assessed as nitrite via the Griess reaction at 550 nm. The supernatant medium (100 µL) was added to 100 µL of a mixture composed of 1% sulfanilamide and 0.1% naphthylethylenediamine dihydrochloride (Sigma-Aldrich), which was incubated for 10 min. The concentration of NO in the culture medium was determined using a standard curve of sodium nitrite (nM/mL), according to Salazar López et al. [34].

### 2.9. Sensory Evaluation

Neither Mexican law nor the institution where the study was conducted require a declaration of ethics in sensory evaluations. Each participant was verbally invited without coercion to participate; the types of food, requirements, and risks of their participation were explained; and the participants could withdraw from the study at any time. During the execution of the study, appropriate protocols were used to protect the rights and privacy of the participants in accordance with the Helsinki Declaration. Oral informed consent was obtained from the panelists.

A sensory evaluation was used to determine the population’s willingness to consume granola bars made with SF, OF, SSF, and ISSF. The sensory test was conducted by untrained members of the general student population (n > 100) of the University of Sonora, Mexico, who responded to a verbal invitation, with no age or gender limits. In each panel, foods made with different flours were evaluated, coded so as not to influence the perceptions of the participants. In each sensory test, samples of bars weighing approximately 47.3 g at room temperature were provided, and the participants evaluated different characteristics of the food using a 9-point hedonic scale. The scale consisted of 1: I dislike it extremely, 2: I dislike it very much, 3: I dislike it moderately, 4: I dislike it slightly, 5: I am indifferent, 6: I like it slightly, 7: I like it moderately, 8: I like it very much, and 9: I like it extremely. This was accomplished by evaluating different characteristics, such as odor, color, taste, texture, and general acceptability to the corresponding food [42].

#### Just-About-Right (JAR) Test

To determine the appropriate levels certain food characteristics, we used the just-about-right (JAR) scale. The attributes evaluated included sweetness, herbal flavor, and firmness. The 5-point scale consisted of 1: too little, 2: very little, 3: just enough, 4: very high, and 5: too high and was used to determine the appropriate levels of the different attributes. At the end of each survey, the participants were asked if they would consume and buy the different types of foods evaluated [43].

### 2.10. Statistical Analysis

In this study, a completely randomized design with three replications for each bar made with different flours was used. Analysis of variance (ANOVA) was performed on the means ± standard deviations. Tukey’s test was used at a significance level of *p* < 0.05. For the analysis of penalties for the levels of attributes in the JAR scale, contingency tables, Chi^2^ tests, Cramer’s V, Pearson’s Phi, and contingency coefficients (C.C) were used to determine the relationships among variables.

## 3. Results and Discussion

### 3.1. Total Phenol Contents and Antioxidant Activities in the Granola Bars

Granola bars are an ideal food for modifying ingredients to develop new products with functionality. An example of this is granola bars enriched with oligofructose from chicory root inulin to treat overweight and obesity in adults [44] and improved bars for hemodialytic patients [45]. Therefore, granola bars with irradiated sorghum sprout flour would be good options as health foods due to the antioxidant potential of their ingredients like oat [46], coconut [47], agave honey [48], cashew [49], and sorghum [50].

**Figure 1:**
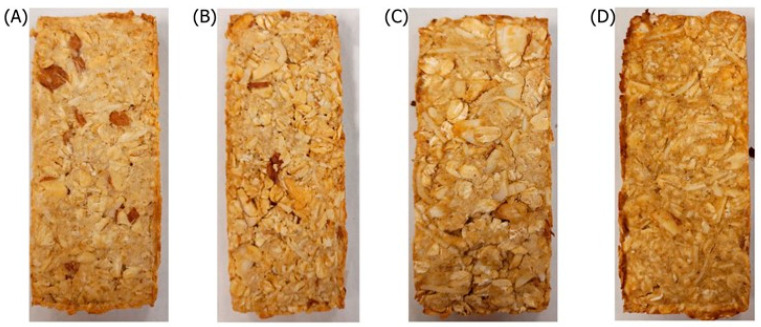
Bars obtained from different types of flour: (**A**) sorghum flour; (**B**) oat flour; (**C**) sorghum sprout flour; (**D**) irradiated sorghum sprout flour.

Among the different granola bars made with flour from sorghum, oats, sorghum sprouts, and ISSF, the bars made with ISSF had the highest total phenol content, which was significantly different from other bars. The total phenol content of the bars made with ISSF was 1.30 times higher than that of the bars made with SSF but 2.43 and 2.90 times higher than that of those made with SF and OF, respectively (see Figure 1).

**Figure 2:**
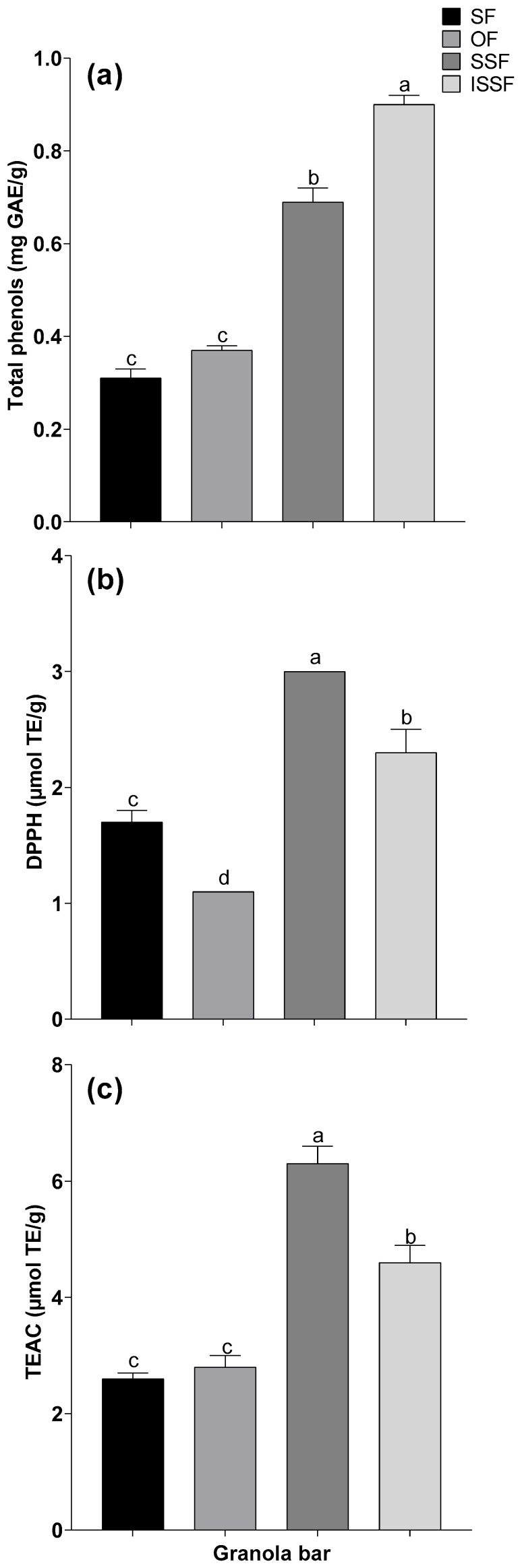
Total phenols and antioxidant activity determined using DPPH and TEAC in granola bars made with different flours. The results are expressed as the means of three replicates. SF: sorghum flour; OF: oat flour; SSF: sorghum sprout flour; ISSF: irradiated sorghum sprout flour. Comparisons were performed via ANOVA, and different letters in the columns of each assay indicate significant differences according to Tukey’s test (*p* < 0.05). (**a**) Total phenols, (**b**) 1,1-diphenyl-2-picrylhydrazyl (DPPH), and (**c**) Trolox equivalent antioxidant capacity (TEAC).

On the other hand, with respect to the antioxidant activity determined using DPPH, granola bars made with SSF had the highest antioxidant activity, which was significantly different from that of the other bars. The antioxidant activity of the bars made with SSF was 1.30 times higher than that of bars made with ISSF, 2.72 times higher than that of bars made with OF, and 1.76 times higher than that of bars made with SF. In contrast, the antioxidant activity of the bars made with ISSF was 2 times higher than that of the bars made with OF and 1.35 times higher than that of the bars made with SF. Similar results were found for antioxidant activity determined using the TEAC, where the antioxidant activity of bars made with SSF was 1.36 times higher than that of bars made with ISSF and 2.4 and 2.2 times higher than that of bars made with SF and OF, respectively. These findings indicate 23% and 26% increases in the antioxidant activity of SSF compared with ISSF, as determined using DPPH and the TEAC, respectively (Figure 2).

As observed, the total phenol content was higher in bars made with ISSF compared to the rest of the bars, but the antioxidant activities determined using DPPH and the TEAC were significantly higher in the bars made with SSF. Since the base ingredients were the same and only the flours used differed, the total phenol content and antioxidant activity did not correlate; these differences could be attributed to two potential factors: the particular antioxidant capacity method used and the interactions between antioxidant compounds. Regarding the method used, it is known that each antioxidant capacity assay has a particular mechanism of action by which it allows the transfer of electrons and/or hydrogen atoms, thereby making some more sensitive for specific molecules and less so for others. Moreover, the behavior of an antioxidant molecule may differ when analyzed individually, as compared to the same molecule in a complex matrix where other antioxidants (and other types of compounds) are also present, potentially leading to synergistic or antagonistic interactions, as observed here. Further analyses may be required to conclusively state if the antioxidant methods and/or molecular interactions are responsible for the observed results.

To understand the increase or decrease in the content of antioxidative compounds in sprouted grains, it is important to remember that unlike in fruits, phenolic compounds in seeds are found mostly in insoluble forms, covalently bound to the cell wall matrix through ester, ether, and C-C bonds [51]. During budding, the structures to which these compounds are bound tend to be hydrolyzed for use in primary plant metabolism and the formation of new structures [52,53]. Under these conditions, those compounds bound to cell walls could be released. Once released, they can act as antioxidants to quench the reactive oxygen species (ROS) generated during metabolic processes enhanced by seed germination, protecting cells from oxidative damage [54,55]. They may also participate in protection against fungal or bacterial infections [56], in the enhancement of antioxidant enzyme activity [57], and in the regulation of the cellular and structural growth of new plants [58].

In contrast, during the elicitation of sorghum grain with UV-A light, metabolic processes such as those mentioned above may be altered, with increases or decreases in said processes to preserve seed integrity [59,60]. The metabolic increase results in an increased release of previously insoluble compounds, as the structures in which they are found are necessary for primary and secondary metabolisms in response to UV stress [61,62]. During these processes, reducing sugars such as glucose, fructose, and sucrose, as well as organic acids and amino acids, are generated or released during sprouting and irradiation [63,64] and can interact with phenolic compounds [65].

On one hand, the differences in the relationship between total phenol content and antioxidant activity may be due to the detection mechanisms and phenolic compounds present in the different flours. For example, the Folin–Ciocalteu assay involves electron transfer; therefore, phenolic acids with various hydroxyl groups, such as gallic acid and protocatechuic acid, are more closely related [66]. On the other hand, detection using DPPH occurs through hydrogen transfer [67], so phenolic acids such as ferulic acid [68] and sinapic acid show greater affinity [69]. For detection using the TEAC, antioxidant compounds act via electron transfer and hydrogen donation [70], so bioactive compounds with conjugated structures and greater redox stability, such as ferulic acid and sinapic acid, are more amenable to the reaction [71,72]. Therefore, the increase in phenols in ISSF bars and their different antioxidant activities could be due to the presence and concentration of certain compounds and the affinity of these compounds for the different techniques used.

Although there is limited information on the use of irradiated sorghum sprouts as possible ingredients for food processing, the use of different sprouts as part of the formation of different functional foods has increased in recent years. Applications for the development of foods based on sprouted grains have already been discussed by Šterna et al. [73], Bazhay-Zhezherun et al. [74], and Finnie et al. [75], so the potential use of these foods is increasing.

### 3.2. Phenolic Acids and Flavonoids

The quantification of total phenols and antioxidant activity is important, and the compounds that can be found include gallic acid, which has demonstrated anti-inflammatory effects, cardiovascular and gastrointestinal protection, and metabolic and neuropsychological effects, among others [76]. Therefore, due to such potential, it is important to identify the types of bioactive compounds present in the different bars. When analyzing bioactive compounds, the chromatographic results indicate the presence of phenolic acids such as gallic acid and protocatechuic acid, among others, and flavonoids such as catechin (Table 2). Gallic acid and catechin were identified in all the bars processed, with the highest concentrations of both compounds being observed in the bars made with ISSF. On the other hand, ferulic and sinapic acids were not identified in any of the processed bars. The results indicate that in the bars made with ISSF, the gallic acid content was 44.75% higher than that in the bars made with SSR. Similarly, the gallic acid content in the bars made with ISSF was 48.9% and 71.8% higher than that in the bars made with SF and OF, respectively. The catechin content in the bars made with ISSF was 1.7% higher than that in the bars made with SSF. Bioactive compounds such as protocatechuic acid and *p*-coumaric acid were not identified in the bars made with ISSF.

Among the bioactive compounds identified, a previous study by Ruiz-Hernandez et al. [77] demonstrated the presence of ferulic acid, sinapic acid, and *p*-coumaric acid in cookies made with ISSF, compounds that were not identified in the bars made with ISSF in this study. However, although ISSF was used for the preparation of the cookies, the formulation of the bars was very different. Furthermore, proportionally, the amount of flour used in the prepared cookies was greater, and the number of basic ingredients was lower and less complex than those used in the bar. For example, among the ingredients, saponins, flavonoids, terpenoids, terpenoids, coumarins, and others have been identified in agave syrup [48]. In coconut oil, gallic acid, epigallocatechin, catechin, *p*-hydroxybenzoic acid, caffeic acid, syringic acid, and ferulic acid were identified by Seneviratne et al. [78]. In contrast, in coconut, gallic acid, caffeic acid, salicylic acid, and *p*-coumaric acid were present [79]. Moreover, luteolin, apigenin, epicatechin, epigallocatechin, and kaempferol can be found in oat [46], cashew nuts [49], and almond [80]; therefore, possible interactions must be considered. Among these possibilities, π–π interactions between the aromatic rings of phenolic compounds could occur [81]. The results found here vary with cookies elaborated on in another study by the total percentage of flour used (57%) and to that used in the bars (9.5%) developed here. This difference and a less complex matrix in the cookies may result in fewer interactions.

On the other hand, it is also important to consider that different interactions of the bioactive compounds present within the food matrix are cumulative. For example, the formation of phenol–protein complexes [82], hydrogen bridges between the hydroxyl (-OH) groups of phenols and the amino groups (-NH₂) of proteins [83], hydrophobic interactions among nonpolar amino acids of proteins [84], and the adsorption of phenolic acids to fibers [85] occur, where polysaccharides present in oats and coconut can trap phenolic acids via hydrogen bonds and Van der Waals forces [86].

Although the different interactions are not clear, the results revealed good concentrations of bioactive compounds. Similar results have been described for the use of sprouts in salable bars with moringa described by Coello et al. [87], in legumes by Rajagukguk et al. [88], and in wheat sprout bars by Yang [89].

### 3.3. Bioaccessibility and Antioxidant Recovery of Phenolic Compounds

The presence of antioxidant compounds in food is very good for health; however, the potential beneficial effect of these compounds is dependent on their release from the food matrix for absorption. This process is called bioaccessibility and is a concept that helps to determine the nutritional efficacy of food formulations developed to improve human health [73]. Therefore, several investigations to measure the potential effect of an ingredient have determined the bioaccessibility of the compound of interest. This has been carried out in assays such as those for sorghum protein [90], hydroxycinnamic acids [40], and starch [91], among others.

**Figure 3:**
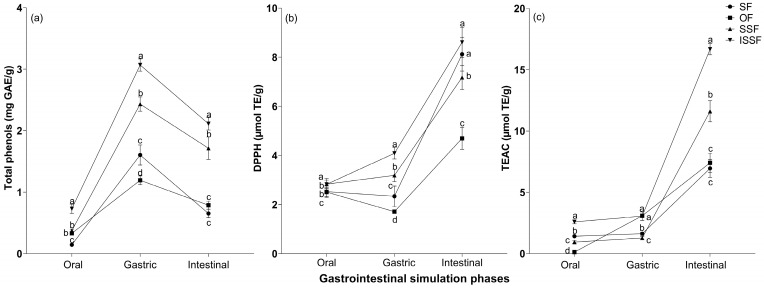
Total phenol content and antioxidant activity of bars made with different flours during the different stages of in vitro digestion. SF: sorghum flour; OF: oat flour; SSF: sorghum sprout flour; ISSF: irradiated sorghum sprout flour. The results are expressed as the means of three replicates. Comparisons were made via ANOVA, and different letters in each digestion phase indicate significant differences between bars according to Tukey’s test (*p* < 0.05). (**a**) total phenols, (**b**) DPPH, and (**c**) TEAC.

Since the beneficial effects of the consumption of healthy foods is limited by their bioaccessibility, this characteristic and the antioxidant recovery of the different bars produced were measured. The results are shown in Figure 3 and demonstrate the dynamic effect of digestion on the phenol content. These results indicate that the bars made with ISSF had the highest phenol concentration and antioxidant recovery compared with the other bars produced, with a ranking of the bars as ISSF > SSF > SF > OF.

In terms of concentration and antioxidant recovery, the concentration of phenols in the bars made with ISSF was 1.4 times higher than that in the bars made with SSF, 3.24 times higher than that in the bars made with SF, and 2.7 times higher than that in the bars made with OF. The antioxidant recovery determined using DPPH was 1.19, 1.06, and 1.86 times higher in the bars made with ISSF than in the bars made with SSF, SF, and OF, respectively. The antioxidant recovery determined via TEAC was several-fold higher in the bars made with ISSF than in the bars made with SSF (1.43), SF (1.67), and OF (2.25) (Figure 2).

When comparing concentrations in phenolic content, the differences that could be observed between digests and undigested feed may be due to several factors related to the release, transformation, and availability of phenolic compounds during digestion [92]. Various phenolic compounds in food are bound to polysaccharides, which can be released by enzymatic action, pH changes, and bile salts [93]. Regarding antioxidant activity, this could be associated with the release of phenolic compounds, as well as with possible interactions between proteins and polyphenols that enhance this capacity [94]. On the other hand, it is possible to consider that in the DPPH assay, overlapping spectra of some compounds that absorb in the same range as the DPPH radical are possible; limitations in TEAC have also been described by failing to evaluate the rate of free radical scavenging [95].

Regarding bioaccessibility and antioxidant recovery from the intestinal fractions of the different bars evaluated (Table 3), the bars made with ISSF presented a high bioaccessibility (119%) and antioxidant recovery (201% for DPPH and 225% for TEAC). With respect to these results, the bioaccessibility of ISSF is 1.3, (SSF), 3.5 (SF), and 2.3 (OF) times higher than the rest of the foods. A similar response was found in antioxidant recovery using DPPH, where ISSF was 3.3 times higher than SSF and 2.1 and 2.2 times higher than SF and OF. Similarly, antioxidant recovery using the TEAC was several times lower in SSF (5.1), SF (5.4), and OF (3.7) compared to ISSF. The higher percentages of bioaccessibility and antioxidant recovery in ISSF compared to the rest of the foods may be due to the higher concentration found (Figure 3) and the possible greater degradation of structural macronutrients of the grains subjected to UV light, resulting in simpler components that favor the release of antioxidant compounds due to the different digestion conditions. It is noteworthy that free, bound, and conjugated phenolics are all considered in the aforementioned bioaccessibility calculation in order to provide a more thorough and comprehensive approach to this variable.

These results provide insight into the potential beneficial effects of consuming these foods and are due to two main factors: First, high concentrations of phenolic compounds were already present in the digested samples, mainly in sprouted (SSF) and sprouted/irradiated samples (ISSF) (see Table 2). Moreover, during digestion, in addition to the interactions of phenolic compounds with the food matrix, chemical transformations, oxidation, polymerization, and degradation can occur [96]. The inhibition of digestive enzymes by the noncovalent binding of some phenolic compounds through electrostatic forces, hydrophobic binding, hydrogen bonding, and Van der Waals forces [97] has been described. Isomerization of some compounds can also occur via structural changes depending on the pH [98], since digestive pH can considerably intensify the antioxidant activity of fiber-rich foods, which could be due to changes in activity and structure [99]. Similar effects could be found in sorghum sprouts, which increased the bioaccessibility of phenols by 10.18% [100].

### 3.4. Phenolic Acids and Flavonoids Found in the Intestinal Fraction

The differences in phenol content and antioxidant recovery may be due to the presence of individual antioxidants released during digestion. When the bioaccessible fraction (intestinal fraction) was analyzed, catechin, sinapic acid, ferulic acid, *p*-coumaric acid, protocatechuic acid, and gallic acid were found in the different bars (Table 4). Among the different samples, significantly higher concentrations of gallic acid, protocatechuic acid, and catechin were detected in the bars made with SSF and ISSF. The bars made with ISSF had a 64.52% higher gallic acid content than did the bars made with SSF. In contrast, the bars made with SSF had a 65.49% higher protocatechuic acid content than the bars made with ISSF. Moreover, the catechin content was 93% higher in the bars made with ISSF than in the bars made with SSF. In comparison, the concentrations of *p*-coumaric acid, ferulic acid, and sinapic acid in the intestinal fraction were low. However, unlike in undigested foods, these were identified in the intestinal fraction during digestion. These results could indicate that due to the digestion conditions, these compounds that could not be released previously were released.

The possible differences found between bars with SSF and ISSF before and after digestion are closely related to the influence of UV light. During the development of the new plant, the degradation of structures during seed germination is an essential process to mobilize reserves and provide energy and nutrients to the growing embryo. This process involves the decomposition of macromolecules stored in reserve tissues by hydrolytic enzymes [53]. On the other hand, the effect of UV-A light can generate oxidative stress, which impacts the synthesis of enzymes related to the mobilization of reserves, which, in turn, causes the release of phenolic compounds until de novo synthesis to alleviate stress [61,101]. Therefore, interactions between complex polysaccharides and proteins are likely to occur in SSF, whereas in ISSF, they would be more common between amino acids, peptides, and monosaccharides, which would explain the dynamic changes between foods and the dynamic effect of simulated digestion.

It is important to note that these compounds can be found in different conformations in the food matrix: they can be found free, forming complexes with other food components; bound to organelles; or trapped in matrices [102]. Therefore, unlike plants, which have specific enzymes to release these compounds for different purposes [103,104], these compounds are released from the matrix during digestion [105]. These compounds are subsequently released from the aforementioned conformations via the effect of the pH, which hydrolyzes covalent and noncovalent bonds, releasing polysaccharides, cell wall structures, proteins, etc. [106]. Similarly, bile salts can destabilize structures in food [107], solubilize lipids and hydrophobic compounds [108], interact with proteins and polysaccharides [93,109], and even interact with various bioactive compounds [110]. Specific enzymes are also responsible for the hydrolysis of proteins, lipids, and polysaccharides [111], which together release trapped bioactive compounds, which, in turn, tend to interact with less complex structures previously hydrolyzed or with the enzymes themselves, decreasing their bioactivity (monosaccharides, amino acids, and fatty acids), which can decrease their bioaccessibility and subsequent absorption [112,113].

Food development involves the use of the same ingredients with some modifications, and these changes offer the population new shapes, textures and flavors. In the previous development of cookies with ISSF, the potential benefits of their consumption were demonstrated by showing the content of bioactive compounds and antioxidants. In contrast, the use of ISSF flour as part of the ingredients for the preparation of granola bars demonstrates the versatility of this product as an ingredient for the formulation of various foods. The content of phenolic compounds before and after gastrointestinal simulation in cookies and bars differs, since proportionally, in the cookie with ISSF, it represents 57% of the total, while only 9.5% of ISSF is present in the processed bars. Thus, a higher content of fiber, proteins, polysaccharides, and different phenolic compounds can be found in the bars, modifying their release, interactions during processing, and digestion, as mentioned above.

### 3.5. Anti-Inflammatory Effects

Inflammation is a physiological defense mechanism, but prolonged inflammation occurs alongside obesity and other conditions, which further promotes negative health effects [114]. Multiple mediators have been associated with chronic inflammation, including tumor necrosis factor α (TNF-α), interleukin [IL]-6, IL-1β, ROS, and chemokines [115]. This inflammatory environment activates inducible nitric oxide synthase (iNOS), which synthesizes NO [116], a known oxidative stress biomarker [117]. Therefore, the effects of food extracts and digests of the intestinal fraction of the different bars were measured to determine their anti-inflammatory potential.

According to the results obtained (Table 5), prior to the inflammation test, the cytotoxic effects of the different extracts were evaluated. High cell viability was observed in response to the granola bars, which did not affect the integrity of the RAW 264.7 cells used. On the other hand, in extracts obtained from the intestinal fraction of the simulated digestion, viability percentages of up to 91.9% were observed in bars made with ISSF at 12.5 µg/g of extract. This means that after treatment, 92% of the cells remained alive and functional, whereas 8% of them died or were damaged; however, they were still considered healthy cultures [118].

It was observed that in the granola bars made with SF at the lowest concentration, there was a greater decrease in NO production, which was significantly different from that in the rest of the foods and concentrations evaluated. There were no significant differences between the different concentrations of bars made with OF, SSF, and ISSF. Different results can be observed for the NO evaluated in the digests of the intestinal fraction; differences are found between the different concentrations of foods, which were lower than those of the positive control. The lowest concentrations of NO were identified in the bars made with ISSF; however, these concentrations were not significantly different from those with 25 µg/mL from the bars made with SSF. While the greatest decrease in NO production was present under these conditions, the concentration necessary to decrease the level of NO production was lower (6.25 µg/g in the bars made with ISSF) than the higher concentrations observed in the bars made with SF and SSF. This finding indicates that a fourfold lower concentration of the extract was required to obtain the same results, which may be due to the presence of higher gallic acid and catechin concentrations in SSF and ISSF, which are able to reduce NO in RAW 264.7 cells. Furthermore, the NO in digests is proportionally reduced to a greater extent than that in undigested foods.

Unlike the evaluation of the anti-inflammatory effects of individual compounds such as quercetin [119], gallic acid [120], or kaempferol [121], where the anti-inflammatory effects can be attributed to these compounds, the use of foods involves understanding the presence and mixture of different antioxidants, whether they are identified or not [122].

For example, although high concentrations of gallic acid and catechin were identified in the bars made with SSF and ISSF and their digests, in general terms, all foods decreased NO production compared with the positive control. However, NO production did not differ greatly between foods, but there were differences in the concentrations in already digested foods. Therefore, it is possible that the maximum effect (threshold of biological activity) was reached, where even the lowest concentration (6.25 µg/mL) was sufficient to achieve a maximum effect on the inhibition of NO production [123]. Additionally, the synergy between compounds must be considered; although high concentrations of gallic acid and catechin exist, other foods could contain other bioactive compounds that also contribute to the reduction of NO. These compounds could act synergistically, resulting in a similar effect at all concentrations tested [124]. In the digests, differences in the NO concentration between foods could be observed, but some previously unidentified compounds were found in the intestinal phase. Therefore, the release of these compounds possibly influences the production of NO. In addition, during digestion, these compounds possibly transform into NO. It should also be mentioned that during digestion, phenolic compounds can undergo transformation to more active metabolites [125], decreasing the activity thresholds. These results support the potential anti-inflammatory effects of these foods, since their concentrations can vary, as can their chemical structure when they are absorbed [126,127]. However, an analysis of other inflammatory biomarkers would be necessary to broaden the perspective of the anti-inflammatory action of processed foods.

### 3.6. Sensory Evaluation, Penalty Analysis, and Consumption Intention

Granola bars can be prepared from different cereals [128], which can be enriched with other ingredients [129]. According to the results of the sensory evaluation, most of the participants evaluating the foods were between 18 and 22 years old (34 men and 64 women), which indicated that they moderately liked the odor, color, flavor, and texture of conventional bars made with OF and that they liked them significantly more than the other prepared bars (*p* < 0.05). These results may be related to the general acceptance of the product, which, according to its Chi^2^ = 0.0001, is significant. The relationship between acceptance and the characteristics evaluated in the OF bar was strong, so the acceptance of this product could be due to the characteristics it presents.

The bars made with SF significantly differed from the bars made with OF; however, the participants only slightly liked the characteristics of the bars made with SF, which was reflected in their general acceptance. This relationship was observed for color, flavor, and texture vs. acceptance, which were significantly related, according to its Chi^2^ = 0.0001. The relationship was medium to strong according to Pearson’s Phi, Cramer’s V, and C.C.

Among the remaining bars, those made with ISSF were acceptable in terms of all characteristics evaluated, which was reflected on the general acceptance with approving responses for this type of bar. These characteristics were significantly related, according to its Chi^2^ = 0.0001, and this relationship was strong according to Pearson’s Phi, Cramer’s V, and C.C. However, the characteristics, as well as the general acceptance, were significantly lower (*p* < 0.05) than those observed for bars made with OF and SF.

**Figure 4:**
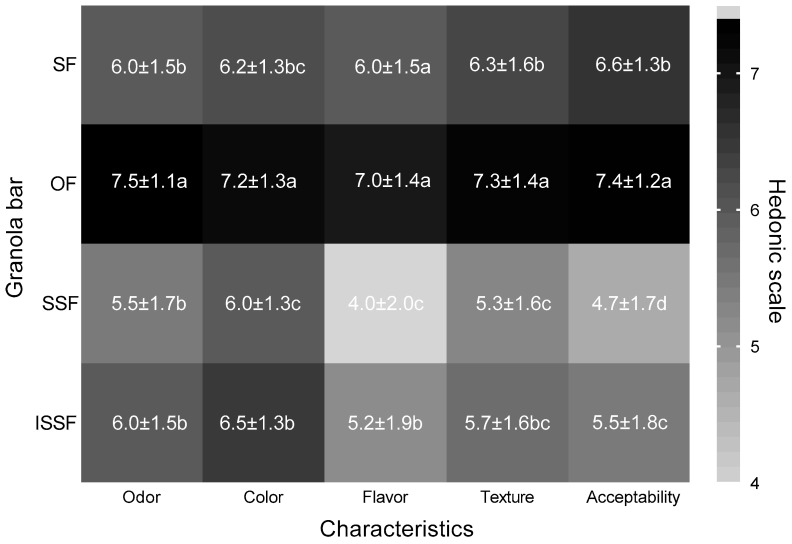
Heatmap of the hedonic scale of bars made with different flours. OF: oat flour; SF: sorghum flour; SSF: sprouted sorghum flour; ISSF: irradiated sprouted sorghum flour. The values of the hedonic scale correspond to 1: dislike extremely, 2: dislike very much, 3: dislike moderately, 4: dislike slightly, 5: neither like nor dislike, 6: like slightly, 7: like moderately, 8: like very much, and 9: like extremely. Comparisons were made via ANOVA; different letters in each column indicate significant differences according to Tukey’s test (*p* < 0.05).

Finally, the bars made with SSF presented lower general acceptance of the product; the results are shown in Figure 4. Some characteristics of these bars, such as their odor, color, and texture, were accepted by the participants, and some were significantly related (Chi^2^ = 0.001) to acceptance. These findings indicate that taste and texture influence the acceptance of bars made with SSF. The general acceptance of the bars was in the order OF > SF > ISSF > SSF, which indicates that the bars made with SF and ISSF had great potential to be well accepted by the population due to their good acceptance here. More than 100 participants were interviewed. The results observed in a sorghum snack bar indicate low acceptance of different sensory characteristics [33]. On the other hand, the mixture of sorghum and cowpea to make bars indicates that the bars made with a greater proportion of sorghum were the least accepted, as previously reported [130].

The low acceptability of treated bars may be due to the high concentration of gallic acid and catechin found, since these compounds have already been identified as causing astringency and bitterness [131]. Likewise, during germination, different volatile compounds characteristic of sorghum can be released, influencing the acceptability of the products [132].

To determine the optimal levels of the attributes (sweetness, herbal flavor, and firmness) that we consider important in the granola bar, the penalty analysis on the JAR scale (Table 6) indicated that, owing to the high herbal intensity of the bars made with SSF, the overall acceptability was penalized by the participants. However, although no penalties were observed in other foods, there were relationships between sweetness and acceptability attributes in bars made with SF according to Chi^2^ = 0.045; this relationship was indicated by Phi = 0.567; Cramer’s V = 0.284; and C. C. = 0.493. Similar results were found for bars made with ISSF, where herbal flavor and acceptability were significantly related (Chi^2^ = 0.001). This relationship may influence product acceptance according to the moderate relationship between these two variables (Phi = 0.798; Cramer’s V = 0.399; C. C. = 0.624).

According to the attributes and characteristics evaluated, when participants were asked about their intention to consume our products, 95% were willing to consume bars made with OF, 83.3% were willing to consume SF bars, 31.4% were willing to consume bars made with SSF, and 50.5% were willing to consume bars made with ISSF. On the other hand, participants were more willing to buy bars made with OF (76.5%) than bars made with SF (50.0%), bars made with SSF (12.7%), or bars made with ISSF (24.0%).

Granola bars are widely consumed; therefore, to improve their characteristics or increase their health benefits, some researchers tend to mix their ingredients with those that have positive effects. It has been shown that considerable percentages of participants are willing to consume granola bars fortified with byproducts obtained from the manufacture of virgin olive oil, possibly because at least 50% of the participants would be willing to consume these foods; this could be due to the complexity of the ingredients, which could improve the characteristics when mixed with the byproducts obtained [133].

## 4. Conclusions

Granola bars made with ISSF presented a higher content of total free phenols; antioxidant activity was greater in bars made with SSF. Gallic acid and catechin were particularly responsive to sprouting and irradiating (SSF and ISSF). The concentration of free phenols and antioxidant activity were higher in the intestinal phase, particularly for the bars made with ISSF. All the bars exerted significant in vitro anti-inflammatory potential. The bars made with SF and OF presented greater acceptability on various specific parameters and overall; the participants did not penalize the overall acceptability with respect to the evaluated attributes, while there was a relationship between acceptability and herbal flavor. Irradiated sorghum sprout bars have great potential as healthy foods due to their demonstrated antioxidant and anti-inflammatory potential. Some limitations are acknowledged, such as using in vitro bioactivity analyses, which could differ from in vivo models; moreover, only young university students evaluated the products, and their opinions and preferences could differ from other populations. Future studies are needed to evaluate different inflammatory biomarkers when consumed in living organisms, as well as possible ingredient reformulation to improve consumer acceptability.

## Figures and Tables

**Table 1:** Formulation of experimental sorghum granola bars.

Ingredient	Experimental Granola Bars			
	SF	OF	SSF	ISSF
Oat flakes(g)	50	50	50	50
Coconut oil (g)	4.3	4.3	4.3	4.3
Agave honey (g)	27	27	27	27
Cashews (g)	12.5	12.5	12.5	12.5
Almonds (g)	12.5	12.5	12.5	12.5
Coconut (g)	12.5	12.5	12.5	12.5
SF (g)	12.5	--	--	--
OF (g)	--	12.5	--	--
SSF(g)	--	--	12.5	--
ISSF(g)	--	--	--	12.5

SF: sorghum flour; OF: oat flour; SSF: sorghum sprout flour; ISSF: irradiated sorghum sprout flour.

**Table 2:** Phenolic acids and flavonoids found in granola bars made from different flours.

Phenols (µg/g)	SF	OF	SSF	ISSF
Gallic acid	627.71 ± 68.47 ^b^	345.50 ± 6.33 ^c^	678.70 ± 8.83 ^b^	1228.55 ± 1.22 ^a^
Protocatechuic acid	25.29 ± 0.47	-	-	-
*p*-Coumaric acid	104.38 ± 0.38 ^a^	16.17 ± 0.45 ^b^	-	-
Ferulic acid	-	-	-	-
Sinapic acid	-	-	-	-
Catechin	25.43 ± 0.15 ^d^	27.81 ± 0.23 ^c^	1202.28 ± 2.02 ^b^	1223.41 ± 2.14 ^a^

SF: sorghum flour; OF: oat flour; SSF: sorghum sprout flour; ISSF: irradiated sorghum sprout flour. The results are expressed as the means of three replicates. Comparisons were performed via ANOVA, and different letters in each row indicate significant differences according to Tukey’s test (*p* < 0.05).

**Table 3:** Bioaccessibility and antioxidant recovery rates of bars made with different flours.

Assay	Bars			
	SF	OF	SSF	ISSF
Total phenols	34.1	51.4	87.9	119.9
DPPH	95.5	87.4	59.2	201.0
TEAC	41.7	60.9	43.9	225.8

SF: sorghum flour; OF: oat flour; SSF: sorghum sprout flour; ISSF: irradiated sorghum sprout flour. All units are expressed as percentages (%) of bioaccessibility of phenolic compounds and antioxidant recovery. Values were calculated as the percentage of the intestinal free fraction with respect to the initial total antioxidant content and activity of the undigested feed (free + bound + conjugated).

**Table 4:** Phenolic acids and flavonoids found in the intestinal fraction digests of granola bars made with different flours.

Phenols (µg/g)	SF	OF	SSF	ISSF
Gallic acid	-	-	658.82 ± 15.05 ^b^	1856.89 ± 7.81 ^a^
Protocatechuic acid	487.01 ± 10.88 ^c^	180.78 ± 7.30 ^d^	2834.01 ± 35.12 ^a^	977.93 ± 6.73 ^b^
*p*-Coumaric acid	53.19 ± 0.63 ^a^	18.23 ± 0.85 ^d^	35.52 ± 3.66 ^c^	40.29 ± 0.89 ^b^
Ferulic acid	50.73 ± 0.34 ^a^	7.65 ± 0.87 ^d^	36.26 ± 0.49 ^b^	7.38 ± 0.76 ^c^
Sinapic acid	-	64.57 ± 2.188 ^c^	89.32 ± 0.91 ^a^	77.79 ± 0.24 ^b^
Catechin	23.11 ± 0.19 ^d^	48.87 ± 0.29 ^c^	100.83 ± 0.11 ^b^	1672.59 ± 4.43 ^a^

SF: sorghum flour; OF: oat flour; SSF: sorghum sprout flour; ISSF: irradiated sorghum sprout flour. The results are expressed as the means of three replicates. Comparisons were made via ANOVA; different letters in each row indicate significant differences according to Tukey’s test (*p* < 0.05).

**Table 5:** Cell viability and inflammation of extracts from prepared bars.

Sample	Concentration (µg/mL)	Cell Viability (%)		Nitric Oxide (NO) (nM)	
		Granola Bar	Intestinal	Granola Bar	Intestinal
LPS+	-	100.1		129.4 ^a^	129.4 ^a^
LPS-	-	108.2		3.6 ^c^	3.6 ^j^
SF	25	98.7	103.6	111.6 ± 2.5 ^bA^	102.8 ± 0.5 ^eB^
	12.5	104.6	102.2	109.9 ± 2.3 ^bA^	105.5 ± 0.7 ^dB^
	6.25	96.8	102.1	108.6 ± 1.5 ^bB^	112.4 ± 0.2 ^bA^
OF	25	100.6	96.9	102.5 ± 1.0 ^bA^	74.7 ± 1.6 ^hB^
	12.5	105.4	103.6	109.7 ± 1.0 ^bA^	96.8 ± 4.4 ^fB^
	6.25	100.5	102.4	112.1 ± 2.1 ^bA^	110.4 ± 0.3 ^cA^
SSF	25	102.5	93.9	107.0 ± 0.4 ^bA^	71.1 ± 3.8 ^hiB^
	12.5	105.4	92.2	111.8 ± 1.5 ^bA^	84.2 ± 5.3 ^gB^
	6.25	100.9	93.5	112.5 ± 0.4 ^bA^	96.7 ± 4.9 ^fB^
ISSF	25	105.1	94.8	109.3 ± 1.1 ^bA^	68.3 ± 1.7 ^iB^
	12.5	105.8	91.9	109.5 ± 2.5 ^bA^	69.9 ± 3.5 ^iB^
	6.25	101.5	107.6	112.5 ± 1.3 ^bA^	74.4 ± 3.9 ^hiB^

SF: sorghum flour; OF: oat flour; SSF: sorghum sprout flour; ISSF: irradiated sorghum sprout flour. The results are expressed as the means of three replicates. Comparisons were performed via ANOVA; different capital letters in each row indicate significant differences between feeds and digests. Lowercase letters in each column indicate differences between the concentrations of each food according to Tukey’s test (*p* < 0.05).

**Table 6:** Penalty analysis of the average decrease in overall acceptance on a 9-point scale and just-about-right (JAR) scores of the granola bars made with different flours.

Variable	Level	OF	SF	SSF	ISSF
Sweetness	Little sweet	−0.085	1.017	0.929	1.336
	JAR				
	Very sweet	0.008	0.817	0.633	1.434
Herbal	Little herbal	0.212	0.495	0.575	0.400
	JAR				
	Very herbal	0.198	0.572	1.300 *	1.600
Firmness	Unsteady	0.421	0.096	0.329	−0.204
	JAR				
	Very firm	0.856	−0.245	0.126	−0.206

An asterisk (*) indicates significant differences (*p* < 0.05).

## Data Availability

The original contributions presented in this study are included in the article. Further inquiries can be directed to the corresponding author.

## Abbreviations

The following abbreviations are used in this manuscript:

ABTS	2,2′-azino-bis(3-ethylbenzothiazoline-6-sulfonic acid
ATCC	American Type Culture Collection
CO_2_	Carbon dioxide
DMEM	Dulbecco’s Modified Eagle Medium
DMSO	Dimethyl sulfoxide
DPPH	1,1-Diphenyl-2-picrylhydrazyl
HCl	Hydrochloric acid
iNOS	Inducible nitric oxide synthase
JAR	Just about right
KCl	Potassium chloride
LPS	Lipopolysaccharide
MTT	3-(4,5-dimethylthiazol-2-yl)-2,5-diphenyltetrazolium bromide
NaOH	Sodium hydroxide
ROS	Reactive oxygen species
TEAC	Trolox equivalent antioxidant capacity
TNF-α	Tumor necrosis factor α
UHPLC-DAD	Ultra-high-performance liquid chromatography diode array detection
UV-A	Ultraviolet light A
